# Malaria around large dams in Africa: effect of environmental and transmission endemicity factors

**DOI:** 10.1186/s12936-019-2933-5

**Published:** 2019-09-03

**Authors:** Solomon Kibret, Jonathan Lautze, Matthew McCartney, Luxon Nhamo, Guiyun Yan

**Affiliations:** 1 Program in Public Health, University of California Irvine, Irvine, CA 92697 USA; 2 International Water Management Institute, Pretoria, South Africa; 3 International Water Management Institute, Vientiane, Laos

**Keywords:** Dams, Malaria, Reservoir shoreline, Slope, Topography, Africa

## Abstract

**Background:**

The impact of large dams on malaria has received widespread attention. However, understanding how dam topography and transmission endemicity influence malaria incidences is limited.

**Methods:**

Data from the European Commission’s Joint Research Center and Shuttle Radar Topography Mission were used to determine reservoir perimeters and shoreline slope of African dams. Georeferenced data from the Malaria Atlas Project (MAP) were used to estimate malaria incidence rates in communities near reservoir shorelines. Population data from the WorldPop database were used to estimate the population at risk of malaria around dams in stable and unstable areas.

**Results:**

The data showed that people living near (< 5 km) large dams in sub-Saharan Africa grew from 14.4 million in 2000 to 18.7 million in 2015. Overall, across sub-Saharan Africa between 0.7 and 1.6 million malaria cases per year are attributable to large dams. Whilst annual malaria incidence declined markedly in both stable and unstable areas between 2000 and 2015, the malaria impact of dams appeared to increase in unstable areas, but decreased in stable areas. Shoreline slope was found to be the most important malaria risk factor in dam-affected geographies, explaining 41–82% (*P* < 0.001) of the variation in malaria incidence around reservoirs.

**Conclusion:**

Gentler, more gradual shoreline slopes were associated with much greater malaria risk. Dam-related environmental variables such as dam topography and shoreline slopes are an important factor that should be considered in efforts to predict and control malaria around dams.

## Background

Rainfall variability disrupts agricultural productivity, contributes to disasters associated with floods and droughts, and hinders economic growth in Africa [1, 2]. To cope with these challenges and provide a platform for advancing water security and sustainable development, the African continent has entered a new era of dam construction [3]. A great number of large and small dams are currently under construction particularly in sub-Saharan Africa (SSA)—the region with the lowest per capita water use in the world [4]. To support Africa’s dam building plans, the World Bank has renewed its financial support over recent years [5].

Dams alter landscapes, increasing the abundance of standing water and drastically changing aquatic ecosystems and the ecological functions associated with rivers. Inevitably these changes result in a range of consequences beyond the direct envisioned benefits of infrastructure development. Dams’ impacts on the transmission of vector-borne diseases such as malaria comprise a particularly pressing public health challenge in Africa. A number of studies document that dams often increase the rate of malaria in communities living close to reservoirs (hereafter referred as reservoir communities), especially in areas of unstable malaria transmission [6–12].

Dams’ impact on malaria typically results from the expansion of habitats suitable for *Anopheles* mosquito breeding. Availability of breeding habitats in the vicinity of dams and the reservoirs they form is often dramatically exacerbated by the expansion of agricultural activities that create numerous breeding habitats for mosquito vectors [12]. Nonetheless, the effect of the expansion of such habitats is context specific since climatic and biophysical factors governing malaria transmission remain important. Rainfall creates breeding habitats that may affect the abundance of breeding sites, even in reservoir communities [13]. Temperature determines the rate of growth of a mosquito’s aquatic stage and the sporogonic development of the malaria parasite inside adult mosquitoes [14, 15]. Similarly, biophysical factors such as geography and land use can influence the suitability of habitats for mosquito development.

Landscape features and ecologic variables have widely been used to explain and predict the spatial variation of malaria incidence in a certain geographical area [16–20]. For instance, several studies indicated that distance to breeding habitats contributed to the heterogeneity in malaria risk across regions [16–18]. In Dakar, Senegal, a study showed that malaria incidence in households within 160 m from a permanent marsh was 74% while the incidence in those located at 900 m were only 17% [18]. Similarly, evidence suggests that village distance to reservoir shoreline and wind direction are significantly correlated with malaria incidence around Ethiopian dams [21, 22]. In the specific context of water storage reservoirs, Jobin [23] suggested that topographical maps may be useful to gauge suitability for vector development along shoreline sites. In controlled laboratory experiments, Endo et al. [24] identified that the steeper the slope of the shoreline, the less suitable they are for vector development. A recent study, noted that the impact of dams on malaria transmission varies in different ecological settings due to local topographic and climatic factors [21].

To better understand the impact of dams on malaria, recent efforts have moved beyond identification of elevated malaria in the vicinity of individual reservoirs to estimate their aggregated contribution to population at risk and disease transmission in Africa [9, 25]. Keiser et al. [9] estimated that 9 million people were at risk of malaria transmission due to large dams in Africa. Kibret et al. [25] conservatively estimated that the presence of 1286 large dams across the region increased the risk for at least 15 million people and were associated with more than 1.1 million additional malaria cases annually. Another recent study further predicted that, without further control measures, transmission around reservoirs could triple by the 2080s as a consequence of climate change and population increases [26].

While efforts to improve understanding of the malaria consequences of large dams mark important progress, past efforts toward Africa-wide analysis have been limited in three ways: (i) reliance on a crude rectangular approximation for reservoir delineation; (ii) assumption that recent levels of malaria transmission are static, whereas actual conditions are dynamic and evolving; and (iii) insufficient focus on the interactions between environmental variables and reservoir presence that influence transmission levels.

In order to devise appropriate malaria control measures, it is crucial to accurately determine the effect of dams on malaria and understand the underlying factors that lead to increased or decreased malaria incidence in their vicinity. This paper reports on the findings of a study to determine the effect of large dams on malaria in sub-Saharan Africa using an approach that incorporated precise reservoir delineation and explicitly considered evolving transmission levels. The study also explored the role of environmental factors in determining the rate of malaria disease incidence in the vicinity of reservoirs.

## Methods

### Study area

This study focused on sub-Saharan Africa (SSA)—geographically, the area of the African continent that lies south of the Sahara Desert. The region accounts for 90% of the global malaria burden where *Plasmodium falciparum*, the most virulent human malaria parasite species, is predominant [27]. Annually, an estimated 174 million malaria cases occur in SSA [28]. However, recent efforts toward increasing provision of bed nets and application of indoor residual spraying resulted in a 40% reduction in clinical malaria cases between 2001 and 2015, and a reduction of 1.6 million malaria deaths in that same period [27].

### Dam and biophysical data

In this article, a large dam is defined as a dam with a height of 15 m or greater from lowest foundation to crest, or a dam between 5 m and 15 m high impounding more than 3 million cubic meters [29]. To obtain locations of large dams, the United Nations Food and Agriculture Organization (FAO) African Dams Database [30] and the International Rivers Database [31], which together contain 1286 georeferenced large dams, were utilized. The accuracy of dam locations was first verified with Google Earth. When the location of a dam did not precisely match the coordinates stipulated in the two databases, manual corrections were made by adjusting the coordinates of a dam to its location as shown in Google Earth. Dams for which precise locations could not be determined, as well as dams without reservoirs (i.e., run-of-river schemes), were removed from the analysis. Ultimately, a total of 884, 892, 907 and 919 large dams in year 2000, 2005, 2010 and 2015, respectively, were included in this study. Locations of more than 80 percent of these dams required correction; in most cases, corrections were minor (< 2 km).

Reservoir perimeters were extracted from the European Commission’s Joint Research Center (JRC) global surface water datasets [32], published through Google Earth Engine. This dataset includes maps of the location and temporal variability in global surface water coverage from 1984 to 2015. The Yearly Seasonality Classification collection contains a year-by-year classification of the seasonality of water based on occurrence values detected throughout the year. The seasonally submerged area for each year between 2000 and 2015 was examined using Google Earth to identify the annual maximum extent of each reservoir each year. These 16 annual maximum water extent layers were jointly examined to determine the maximum extent of the reservoirs. The 16-year (2000–2015) permanent water extent layer was also used to determine the areas permanently submerged throughout the16 years. The difference between maximum and permanent water surface area was estimated to calculate ‘seasonally submerged area’ for each of the large dams. These data were imported to ArcGIS.

Although JRC Monthly Water Classification History v1.0 dataset contains maps of the location and temporal distribution of surface water from 1984 to 2015, some reservoirs were partially or fully not covered by the respective monthly surface water layer in certain months due to cloud cover or unavailability of Landsat satellite images; a “0-no data” value displayed in images to represent those areas. When area of “0-no data” was less than 30% of maximum surface water area of a reservoir, monthly surface area was extracted from the layer and it was used in our analysis. When “0-no data” was greater than 30% of maximum surface water area of a reservoir, it was recorded as “No Sufficient Data” and removed from analyses.

The elevation and slope of reservoir shorelines were extracted from the Shuttle Radar Topography Mission (SRTM) database [33]. SRTM was flown aboard the space shuttle Endeavour February 11–22, 2000. This mission used single-pass interferometry, which acquired two signals at the same time by using two different radar antennas. Differences between the two signals allowed the calculation of surface elevation. Endeavour orbited Earth 16 times each day during the 11-day mission, completing 176 orbits over 80% of the Earth’s land surface between 60° north and 56° south latitude with data points posted every 1 arc-second (approximately 30 m). This SRTM 30 m digital elevation model (DEM) was used to determine the elevation of each reservoir at maximum water level.

Slope is one of the important topographic parameters that could influence the abundance and suitability of puddles that serve as mosquito larval habitat. It is defined as the rate of change of elevation of the land per unit distance. To determine the slope of the draw-down zone of each reservoir, the seasonally-submerged areas were determined by comparing the maximum extent of the reservoir area with the permanently-submerged area, using data from the European Commission’s Joint Research Center (JRC) [32]. The shape of the seasonally-submerged area was demarcated and overlaid on the DEM. The slope was calculated as the average slope over the entire area of the seasonally submerged part of each reservoir. The slope was not calculated by using the altitude values at maximum and permanent water levels. Instead, the DEM provided altitude data at pixel level (30 m spatial resolution). The pixel-wise slope was calculated from SRTM 30 m DEM. The slope of each pixel is calculated using the altitude values of that pixel and its eight surrounding neighbouring pixels. These pixel-wise slope values were averaged for the seasonally submerged areas to get the average slope of each reservoir. Average slope was treated as the average of the DEM pixels comprising the seasonally-submerged area.

### Malaria, population and climate data

Data for vector distribution were obtained from the Malaria Atlas Project (MAP) database [34]. The MAP database contains a georeferenced illustration of the major malaria vector species in different malaria-endemic areas in Africa. It comprises a combination of aggregated raw data and modelled data to in-fill. It is widely used for regional scale analyses [9, 25, 26] since real field data at the continent scale are not available.

Annual malaria incidence data were obtained from the MAP database [27]. Data were acquired for the years 2000, 2005, 2010 and 2015. These years were selected to align with updates to Worldpop population data [35], which are recomputed every 5 years. MAP produced a 1 km resolution map of annual malaria incidence from Africa based on 33,761 studies across the region. These data were imported to ArcGIS for analysis. Annual malaria incidence was calculated as the number of clinical malaria cases per 1000 person-year at risk. To ascertain the impact of dams on malaria incidence rates as a function of distance, four distance zones were created: 0–1 km, 1–2 km, 2–5 km and 5–10 km (control) relative to the highest water level of the reservoirs. Populations residing more than 5 km from a reservoir perimeter were considered as not at risk from dam induced malaria transmission since the maximum mosquito flight range is considered to be 5 km [36]. The 5–10 km zone served as a control.

Annual population data of SSA were obtained from the Worldpop database [35]. A 1 × 1 km gridded population map was imported to ArcGIS for analysis. The total number of people living in each distance zone was determined for each reservoir every 5 years for the period 2000–2015.

Mean annual rainfall and minimum temperature data were used for climate analyses. Rainfall data were abstracted from the Climate Hazards Group InfraRed Precipitation with Station (CHIRPS) database—which is a 30+ year quasi-global rainfall dataset [37]. CHIRPS incorporates 0.05° (approximately 5 km) resolution satellite imagery with in situ station data to create a 5 × 5 km gridded rainfall time series. Mean minimum surface air temperature data were abstracted from the GHCN CAMS high-resolution (0.5 × 0.5°) global land surface temperature database. Monthly data (in °C) were used to calculate the mean annual minimum temperatures for each year and data were then imported to ArcGIS.

The same projected coordinate system was used throughout the whole analysis for the whole continent (WGS 84 Web Mercator). All the datasets used were from the same coordinate system. Most datasets in this study (MAP, Population, Climate) come in WGS 84 format which simplified the analyses.

### Data analysis

To illustrate the locations of reservoirs with respect to different *Anopheles* species, malaria vector distribution obtained from the MAP database [34] was overlaid on the SSA’s dam–malaria map to show the risk of malaria transmission around reservoirs in areas of different vector composition.

Areas were categorized as stable (> 0.1 malaria cases per 1000 person-year), unstable (≤ 0.1 cases per 1000 person-year) and no malaria (zero malaria incidence) based on the level of malaria incidence in each of four years: 2000, 2005, 2010, and 2015 [38]. The number of dams in each of the three stability categories for each of the four years was determined, as well as the population at-risk (< 5 km of reservoir shorelines) of dam-related malaria.

The number of annual malaria cases was estimated for each distance zone (0–1 km; 1–2 km; 2–5 km and > 5 km) by multiplying malaria incidence rates by the population in each zone. A non-parametric Mann–Whitney test was applied to determine differences in malaria incidence among the four zones.

The annual number of malaria cases associated with dams was determined for areas of both unstable and stable transmission. The number of annual malaria cases attributable to dams was calculated as (I_1_–I_2_) P, whereas I_1_ and P are malaria incidence and total human population in communities living within 5 km of reservoirs, and I_2_ is malaria incidence in communities living between 5 and 10 km from reservoirs.

Relative risk (RR) was used to compare annual malaria incidences in communities close to (< 5 km) and further from (5 to 10 km) reservoir shorelines in both stable and unstable areas in different years. It is calculated by dividing malaria incidence in < 5 km by incidence in 5–10 km as follows:

$${\text{RR}} = {{\left( {\frac{{{\text{No}}.\,{\mkern 1mu} {\text{annual malaria cases in}} < 5\;{\text{km}}}}{{{\text{No}}.\,{\mkern 1mu} {\text{people living in}} < 5\;{\text{km}}}}} \right)} \mathord{\left/ {\vphantom {{\left( {\frac{{{\text{No}}.\,{\mkern 1mu} {\text{annual malaria cases in}} < 5\;{\text{km}}}}{{{\text{No}}.\,{\mkern 1mu} {\text{people living in}} < 5\;{\text{km}}}}} \right)} {\left( {\frac{{{\text{No}}.\,{\mkern 1mu} {\text{annual malaria cases in}} < 5{-}10\;{\text{km}}}}{{{\text{No}}.\,{\mkern 1mu} {\text{people living in}} < 5{-}10\;{\text{km}}}}} \right)}}} \right. \kern-\nulldelimiterspace} {\left( {\frac{{{\text{No}}.\,{\mkern 1mu} {\text{annual malaria cases in}} < 5{-}10\;{\text{km}}}}{{{\text{No}}.\,{\mkern 1mu} {\text{people living in}} < 5{-}10\;{\text{km}}}}} \right)}}$$

To determine any correlation between environmental variables and malaria incidence around dams (< 5 km) in stable and unstable areas, univariate associations were first examined by regressing single explanatory factors (i.e. environmental variables) against malaria incidences. The cross-correlation association between the independent variables was tested for collinearity (see Additional file 1) and the results showed that correlation coefficients were < 0.6 for all variables. Those variables with significant (*P *< 0.05) relationship with the dependant variable in univariate regression were further analysed using multivariate linear regression to identify the environmental factors that best explained malaria incidence in each region. Only those variables with a significant correlation (*P* < 0.05) with malaria incidence were added in the multiple regression models. All variables were tested for normal distribution before analysis. All variables were normally distributed except annual malaria incidence and slope. Nonparametric Mann–Whitney *U* test was applied to determine differences in malaria incidence between stable and unstable areas as well as between reservoir and non-reservoir communities. All analyses were done using Microsoft Excel and SPSS version 22.

## Results

**Fig. 1:**
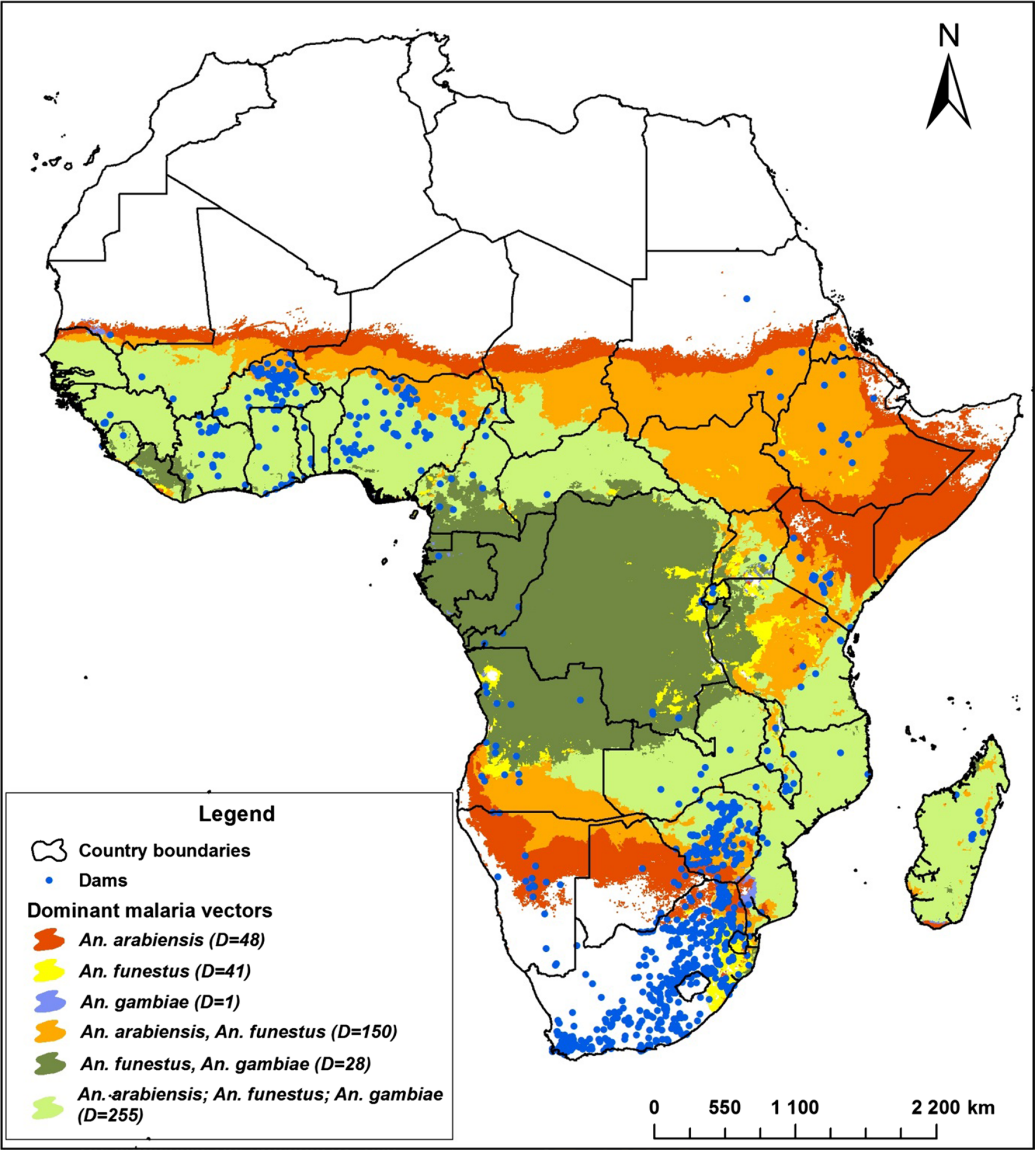
Distribution of dams with respect to major malaria vectors across sub-Saharan Africa

*Anopheles arabiensis, Anopheles funestus* and *Anopheles gambiae* are the major vectors around large dams in SSA (Fig. 1). *Anopheles arabiensis* frequently predominate in the vicinity of dams in unstable areas, whereas *An*. *arabiensis*, *An*. *gambiae* and *An*. *funestus* often coexist in the vicinity of dams in stable areas.

Overall, the population living close to (i.e., < 5 km) large dam reservoirs in SSA increased by about 4.3 million (i.e., from 14.4 million to 18.7 million) between 2000 and 2015 (Table 1). During this period, the number of dams for which geo-referenced data were available increased from 884 in 2000 to 919 in 2015. A total of 18.7 million people lived close to the large dams investigated in 2015. Nearly 75% of the population resides in malarious areas. There was a substantial increase in the population in unstable areas where the number of people living around the dams doubled between 2000 and 2015, as a result of both growing population and growing area of unstable transmission. Nonetheless, large dams in areas of stable transmission tend to be located in more densely populated regions, resulting in greater population at risk around dams in areas of stable transmission.

**Table 1:** Summary of number of dams, area and population living close to large dams in stable, unstable and no malaria areas in sub-Saharan Africa

Year	Stable	Unstable	No malaria	Total
2000				
No. dams (%)	264 (29.9)	199 (22.5)	421 (47.6)	884 (100)
Area (km^2^)	13,617, 900	5,682,270	14,138,000	33,438,170
No. population (%)	7,964,430 (55.3)	2,588,419 (17.9)	3,870,794 (26.8)	14,423,643 (100)
2005				
No. dams (%)	258 (28.9)	214 (24.0)	420 (47.1)	892 (100)
Area (km^2^)	12,503,300	6,800,650	14,134,400	33,438,170
No. population (%)	8,228,448 (52.4)	3,292,887 (21.0)	3,870,794 (26.8)	14,423,643 (100)
2010				
No. dams (%)	249 (27.5)	238 (26.2)	420 (46.3)	907 (100)
Area (km^2^)	9,428,080	9,875,990	14,134,300	33,438,170
No. population (%)	9,757,276 (53.4)	4,044,075 (22.1)	4,475,911 (24.5)	18,277,262 (100)
2015				
No. dams (%)	234 (25.5)	265 (28.8)	420 (45.7)	919 (100)
Area (km^2^)	8,504,050	10,800,100	14,134,300	33,438,170
No. population (%)	8,302,235 (44.4)	5,652,124 (30.2)	4,762,324 (25.4)	18,716,683 (100)

**Fig. 2:**
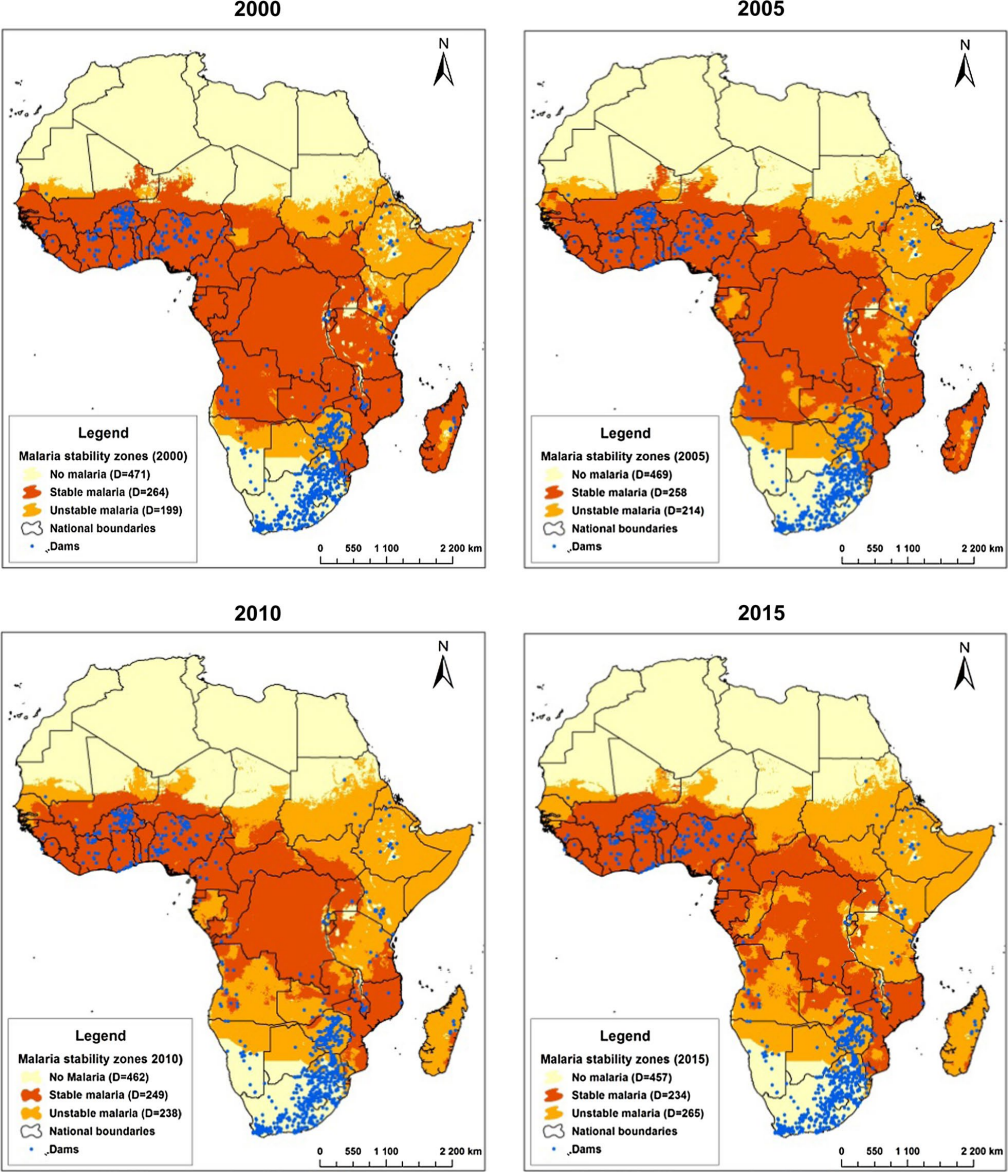
Temporal distribution of dams in relation to malaria stability in sub-Saharan Africa

Overall, recent years have seen a growing number of dams situated in areas with unstable transmission (Fig. 2). The number of dams situated in areas of stable transmission reduced from 264 in 2000 to 234 in 2015 while the number of people living close to these reservoirs increased from 7.9 million to 8.3 million (Table 1). The number of dams located in unstable areas grew during the same period (199 in 2000 and 265 in 2015; a 33% increase), as did the population living close to them (2.6 million in 2000 to 5.6 million in 2015; a 218% increase). In contrast, the number of dams situated in no malaria areas remained virtually the same between 2000 (n = 421) and 2015 (n = 420).

Annual malaria incidence has declined markedly between 2000 and 2015 in both reservoir and control communities (Table 2). In communities close to (< 5 km) large reservoirs in stable areas, incidence decreased from 43.3 cases per 1000 person-year to 29.7 cases per 1000 person-year between 2000 and 2015 while the absolute number of cases increased from just under 3.5 million in 2000 to just over 3.5 million in 2015. In communities close to (< 5 km) reservoirs in unstable areas, the incidence of malaria roughly halved from just over 25 cases per 1000 person-year in 2000 to just under 12 cases per 1000 person-year in 2015 while the absolute number of cases increased from just over 481,000 in 2000 to just over 639,000 in 2015. Absolute numbers of malaria cases increased over this period, due mainly to population growth.

**Table 2:** Annual malaria incidence (expressed as the number of clinical cases per 1000 person-year) and number of annual malaria cases around large dams in sub-Saharan Africa

	Distance cohort (km)	2000		2005		2010		2015	
		Malaria incidence	No. cases	Malaria incidence	No. cases	Malaria incidence	No. cases	Malaria incidence	No. cases
Stable	< 5	43.3*	3,448,500	42.4*	3,485,490	39.3*	3,832,743	29.7*	3,511,483
	0–1	52.1	543,332	50.6	595,494	45.1	593,448	36.8	453,107
	1–2	48.3	731,648	47.1	749,875	40.6	796,294	31.5	563,976
	2–5	40.2	2,173,520	39.2	2,140,121	37.7	2,443,001	28.3	2,494,400
	5–10	25.2	3,708,154	34.1	3,921,842	31.1	3,899,928	22.4	3,492,512
Unstable	< 5	25.4	481,626	19.1	473,660	14.1	486,790	11.7	639,143
	0–1	32.8^#^	136,061	26.9^#^	122,680	19.2^#^	113,286	16.9^#^	152,670
	1–2	29.8	113,619	20.1	91,603	16.5	104,727	12.8	148,981
	2–5	21.1	231,946	16.6	259,376	12.1	268,777	9.9	337,492
	5–10	16.2	419,324	11.8	388,561	9.4	380,143	6.1	344,780

* In stable area, annual malaria incidence rate differenced significantly in communities < 5 km and 5–10 km from the dam for all years (Mann–Whitney test, *P *< 0.05 for all comparisons after the Bonferroni corrections for multiple comparisons)

^#^In unstable area, annual malaria incidence differed significantly among the four distance cohorts for all years (Mann–Whitney test, *P *< 0.05 for all comparisons after the Bonferroni corrections for multiple comparisons)

**Fig. 3:**
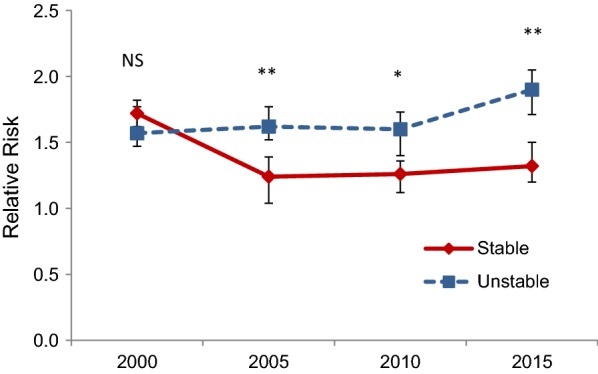
Relative risk of annual malaria incidence in communities close to (< 5 km) reservoir shorelines to those living farther away (5–10 km). The vertical bar indicates 95% CI. *NS* non-significant, *P < 0.05, **P < 0.001 for comparison of the relative risk of communities in stable area with those in unstable area

Against a background of declining incidence, the impact of dams appears to decrease in stable areas and increase in unstable areas, between 2000 and 2015 (Fig. 3). In stable areas, in 2000, the average annual malaria incidence in communities close to (< 5 km) reservoir shorelines were more than 1.7 times higher (*P *< 0.05) than communities farther away (5–10 km). In 2015, the Relative Risk (RR) of malaria incidence in communities living close to the reservoirs (< 5 km) was only about 1.3 when compared to those living farther away (*P *= 0.05). By contrast, in unstable areas, analogous RR increased from just over 1.5 in 2000 to more than 1.9 in 2015 (*P* < 0.05).

**Fig. 4:**
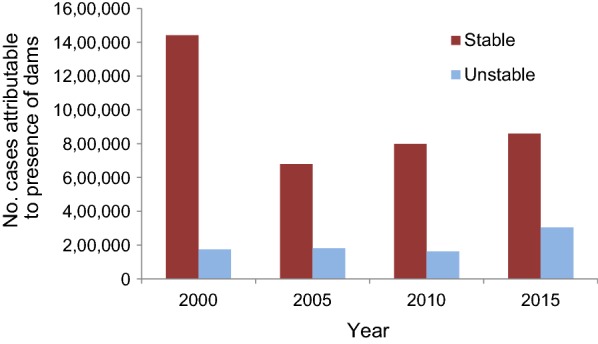
Number of annual malaria cases attributable to presence of dams in stable and unstable areas of sub-Saharan Africa

The absolute number of malaria cases associated with large dams was more than 1.6 million in 2000. The magnitude of impact may partially result from relatively low transmission in control communities in 2000. Subsequent years followed a more logical trend: 679,000 cases in 2005, 798.000 cases in 2010, and 1.2 million cases in 2015 (Fig. 4). The majority of cases were found around dams in stable areas, due to the greater population and higher incidence in these areas.

Different environmental factors were assessed to determine their relationship with malaria transmission around dams (Table 3). The correlation between malaria incidence and slope and seasonally-submerged shoreline area were highly significant (*P *< 0.001). Rainfall and minimum temperature were also significantly (*P *< 0.05) correlated with malaria incidence. Slope had the highest negative correlation; the steeper the slope, the lower the malaria incidence.

**Table 3:** Univariate correlation between environmental factors and malaria incidence in communities living in < 5 km and further away from the reservoirs

Factors	Stable area		Unstable area	
	Pearson’s correlation	*P*	Pearson’s correlation	*P*
Dam communities (< 5 km)				
Slope (°)	− 0.48	< 0.001	− 0.73	< 0.001
Elevation (m)	− 0.26	< 0.05	− 0.56	< 0.001
Receding shoreline area (m^2^)	0.39	< 0.001	0.45	< 0.001
Rainfall (mm)	0.25	< 0.05	0.37	< 0.05
Minimum temperature (°C)	0.27	< 0.05	0.39	< 0.05
Humidity (%)	0.16	> 0.05	0.28	< 0.05
Non-dam communities (5–10 km)				
Slope (°)	− 0.41	< 0.001	− 0.64	< 0.001
Elevation (m)	− 0.32	< 0.05	− 0.59	< 0.001
Receding shoreline area (m^2^)	0.19	> 0.05	0.16	> 0.05
Rainfall (mm)	0.48	< 0.001	0.67	< 0.001
Minimum temperature (°C)	0.22	< 0.05	0.37	< 0.05
Humidity (%)	0.18	> 0.05	0.39	< 0.05

Slope alone was found to be the most important factor explaining 46.8% (*P *< 0.001) of the variation in malaria incidence around large dams (Table 4). A unit degree increase in slope was associated with a 9.4 and 12.5 unit decrease in malaria incidence in stable and unstable areas, respectively. Slope, rainfall and minimum temperature together explained 74.2% and 81.3% of the variation in malaria transmission around reservoirs in stable and unstable areas, respectively.

**Table 4:** Multivariate regression analysis between annual malaria incidence and environmental factors

Area	Model	Standardized coefficient (95% CI)			Adjusted R^2^	*P*
		Slope	Rainfall	Temperature		
Stable malaria	1	− 7.4 (− 5.6, − 9.2)			0.41	< 0.001
	2	− 3.6 (− 3.2, − 4.1)	4.6 (3.9, 5.3)		0.55	< 0.001
	3	− 4.2 (− 3.1,− 5.3)	3.5 (2.8, 4.2)	2.4 (1.8, 3.0)	0.74	< 0.001
Unstable malaria	1	− 12.5 (− 11.8, − 13.2)			0.48	< 0.001
	2	− 8.4 (− 6.5, − 10.3]	6.2 (4.4, 8.1)		0.56	< 0.001
	3	− 6.6 (− 6.0, − 7.2)	4.1 (3.2, 5.0)	3.6 (2.3, 4.9)	0.81	< 0.001

Dependent variable was annual malaria incidence in this analysis

**Fig. 5:**
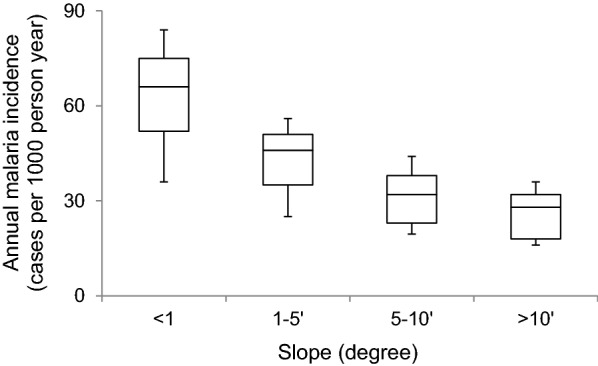
Box plot of malaria incidence against reservoir slope (the boxes show the 25th percentile, median and 75th percentile, and value ranges)

Gentler, more gradual slopes in the seasonally-submerged areas of reservoirs are associated with much greater malaria transmission (Fig. 5). Considering communities around dams (< 5 km) in both stable and unstable zones, malaria incidence dropped from 68 cases per 1000 person-year to 45 cases per 1000 person-year when slope increased from < 1° to 1–5°. Slopes between 5 and 10°, and greater than 10°, were associated with around 30 cases per 1000 person-year. Ultimately, therefore, slopes less than 5 degrees, and especially less than 1° seem extremely conducive for malaria transmission.

## Discussion

In the present study, the findings indicated that 659,000–1.6 million annual malaria cases were attributable to large dams and that 14.4–18.7 million people were at risk of contracting malaria due to large dams in Africa each year. These numbers generally trended upwards over the years of analysis. The study also found that the relative impacts of large dams in areas of unstable transmission are not just statistically greater but such impacts are trending upwards even more than in stable areas. Finally, the present findings reveal that topography around a specific dam site (slope) is the most important factor affecting malaria incidence. The steeper the slope the lower the chance to create transient shallow puddles for mosquito breeding. Reservoir water levels typically fluctuate through a year and during periods of drawdown (i.e. falling water-levels) flat shorelines often create numerous long-lived puddles disconnected from the main water body, which are often ideal for mosquito breeding [12].

Consistent with broader evidence on the results of the Roll Back Malaria (RBM) program in Africa [28], the present study highlighted how malaria incidence is decreasing in both dam and non-dam communities. Unlike previous work [25], the results herein point to a greater aggregate effect of dams in areas of stable transmission, due to the considerably greater population in such areas. Generally, the impact of dams on relative risk of malaria tends to be greater as transmission levels decrease. According to the World Health Organization report [27], annual malaria incidence decreased by 52% between 2000 and 2015. Around African dams, a similar magnitude reduction of malaria incidence was estimated during the same period in unstable areas (54%) but only a 31% reduction was estimated in stable areas. Intensive malaria control is thus required to reduce the malaria impact of dams in stable and unstable areas.

The present study found a growing number of dams in areas with unstable transmission while the number of dams situated in areas of stable transmission reduced between 2000 and 2015. The changing estimates of number of dams in different transmission zones evidenced between 2000 and 2015 is explained primarily by shifting boundaries of malaria transmission stability. Changes in boundaries of stability may result from improved malaria control efforts and climate changes that have lowered transmission in some areas such that they have moved from stable to unstable transmission. Ultimately, expansion of unstable areas and contraction of stable areas has resulted in an increase in the number of dams in unstable areas.

Juxtaposing central findings from this study—(i) rates of malaria are declining, near and far from dams, (ii) the number of dams in unstable zones of malaria transmission is increasing, and (iii) the relative impact of dams in areas of unstable transmission intensifies with decreased transmission—suggests that dams may pose a significant barrier to malaria reduction and eradication in Africa. Indeed, it would appear that the more progress is made to reduce malaria transmission through national, regional and international programs, the more the confounding impact of dams will become. Therefore, intense, direct focus on reducing dams’ impacts is called for, and the time for sidelining dams’ impacts as a relatively peripheral issue to malaria control may soon be over.

Direct focus on dams’ impacts likely requires a central focus on reservoir shoreline slope. Hydrologically, slope acts as a proxy for the likelihood of an area to retain surface water. Gentle slope generally corresponds to poor drainage, thereby promoting persistence of surface water bodies and the formation of stable pools convenient for mosquito breeding. In contrast, steeper slope facilitates drainage and reduces the likelihood that pools will form for periods of sufficient duration for mosquitoes to complete their aquatic stages. The findings presented in this paper are consistent with scant evidence from elsewhere. A study in Zambia [39] showed a small change in slope affects the risk of malaria; households with malaria positive residents were on flatter areas (slope of 0.024° ± 0.014°) than malaria negative households (slope of 0.031° ± 0.023°; *P *= 0.04). In western Kenya, valleys with gentle slopes were characterized by slow moving rivers, poor drainage and with large surfaces to hold water suitable for larval breeding while valleys with steeper slopes were characterized by fast running rivers in the valley bottoms, that were unsuitable for mosquito breeding [40]. Jobin [23] acknowledged the importance of the shoreline topography on malaria transmission around dams as gauged from professional judgement, and Endo et al. [24] explored this issue in laboratory experiments.

Reservoirs also affect the groundwater table. The presence of the reservoir induces higher groundwater levels close to the reservoir than further away. Surface pools that can serve as mosquito breeding habitats tend to be stable in areas where drainage may be inhibited because the groundwater level is high and close to the surface. Depending on micro-topography, sometimes groundwater will seep into depressions creating longer-lived, semi-permanent pools [40]. Such stable habitats are often rich in nutrients and maybe partly covered by vegetation thereby creating ideal breeding habitat for malaria mosquitoes. Moreover, agricultural practices are more common on gently sloping shorelines and such activities tend to increase the availability of breeding habitat for malaria mosquitoes. For example, livestock grazing on the shoreline of the Koka reservoir in Ethiopia created numerous water filled hoof prints: ideal habitat for mosquito larvae [12].

The findings regarding shoreline slope are particularly relevant for impact assessment, mining water storage facilities and environmental control dams. Scuddler [41] indicated the critical need for impact assessment studies to determine social, environmental and institutional costs of large dams. The present work further adds the public health challenges associated with large dams if left unchecked during planning and implementation.

The present study has several limitations. First, average shoreline slope was used in our calculations. In reality shoreline slope will vary around a reservoir and may affect where communities are located which will in turn affect the overall impact of any dam. Second, 25% of the existing supposedly georeferenced dams could not be correctly located so were excluded in this study. Overall, application of a rigorous approach for reservoir delineation led to substantial modifications to dam locations and considerable reductions in the number of dams utilized relative to previous work [25]. Considering that the initial set of georeferenced dams represents only about two-thirds of the total number of dams that are known to exist in Africa [29], findings generated from this work may ultimately comprise less than half of the total number of currently existing large dams in Africa. The impacts identified herein, therefore, reflect a conservative estimation of large dams’ impact. Third, MAP data fills in “no-data” areas with model extrapolation using, amongst others, rainfall and temperature data. Thus, it is not surprising that our results showed significant correlation with these variables. However, slope is not an environmental factor used in the MAP modelling, so the fact that slope emerged as such a strong explanatory variable for variation in malaria incidence—despite potentially inflated model predisposition toward climate variables—underlines the significance of this paper’s major finding. Because this study was a statistical investigation, rigorous exploration of the biophysical processes that explain these relationships should be the central focus of follow-on work.

## Conclusion

As global malaria efforts continue to shrink malaria distribution, dam associated reservoirs in sub-Saharan Africa will continue to offer foci for malaria transmission. In particular, large reservoirs with shallow shoreline slope seem to facilitate malaria transmission by enhancing breeding habitat for malaria vector mosquitoes. This calls for more rigorous impact assessments to determine likely malaria impacts while planning African dams. Explicit consideration of the presence and degree of topographic characteristics at potential dam sites could enable water managers, in collaboration with health planners, to better address the pervasive water–malaria challenge.

## Supplementary information

**Additional file 1.** Cross-correlation of environmental variables (values shown are r values).

## Data Availability

The raw data will be made available upon request as the study is ongoing.

## Abbreviations

CHIRPS: Climate Hazards Group InfraRed Precipitation with Station
DEM: digital elevation model
FAO: United Nations Food and Agriculture Organization
JRC: European Commission’s Joint Research Center (JRC)
MAP: Malaria Atlas Project
RR: relative risk
SRTM: Shuttle Radar Topography Mission
SSA: sub-Saharan Africa
