# Supplementary material for: Malaria around large dams in Africa: effect of environmental and transmission endemicity factors
Source: Malar J. 2019 Sep 3;18:303. doi: 10.1186/s12936-019-2933-5 (PMC6720395; doi:10.1186/s12936-019-2933-5)
Supplement: Supplementary file 1 — Additional file 1. Cross-correlation of environmental variables (values shown are r values). [file 12936_2019_2933_MOESM1_ESM.docx]

Additional file 1: Cross-correlation of environmental variables (values shown are r values)

|  | Slope (^o^) | Elevation (m) | Receding shoreline area (m^2^) | Rainfall (mm) | Minimum temperature (^o^C) | Humidity (%) |
| --- | --- | --- | --- | --- | --- | --- |
| Slope (^o^) |  | 0.45 | -0.32 | 0.08 | 0.11 | 0.16 |
| Elevation (m) | 0.45 |  | -0.18 | 0.26 | -0.31 | 0.13 |
| Receding shoreline area (m^2^) | -0.32 | -0.18 |  | -0.29 | 0.07 | 0.24 |
| Rainfall (mm) | 0.08 | 0.26 | -0.29 |  | -0.12 | 0.11 |
| Minimum temperature (^o^C) | 0.11 | -0.31 | 0.07 | -0.12 |  | 0.22 |
| Humidity (%) | 0.16 | 0.13 | 0.24 | 0.11 | 0.22 |  |
|  |  |  |  |  |  |  |
